# Effectiveness of a Government-Organized and Hospital-Initiated Treatment for Multidrug-Resistant Tuberculosis Patients-A Retrospective Cohort Study

**DOI:** 10.1371/journal.pone.0057719

**Published:** 2013-02-25

**Authors:** Pei-Chun Chan, Su-Hua Huang, Ming-Chih Yu, Shih-Wei Lee, Yi-Wen Huang, Shun-Tien Chien, Jen-Jyh Lee, and the TMTC

**Affiliations:** 1 Third Division, Centers for Disease Control, Taipei, Taiwan; 2 Department of Internal Medicine, Taipei Medical University-Wan Fang Hospital, Taipei, Taiwan; 3 Pulmonary and Critical Care Unit, Tao-Yuan General Hospital, Department of Health, Tao-Yuan, Taiwan; 4 Pulmonary and Critical Care Unit, Chang-Hua Hospital, Department of Health, Chang-Hua, Taiwan; 5 Pulmonary and Critical Care Unit, Chest Hospital, Department of Health, Tainan, Taiwan; 6 Department of Internal Medicine, Buddhist Tzu Chi General Hospital, Tzu Chi University, Hualien, Taiwan; Instituto de Higiene e Medicina Tropicalm, Portugal

## Abstract

**Background:**

In contrast to the conventional model of hospital-treated and government directly observed treatment (DOT) for multidrug-resistant tuberculosis (MDR-TB) patient care, the Taiwan MDR-TB Consortium (TMTC) was launched in May 2007 with the collaboration of five medical care groups that have provided both care and DOT. This study aimed to determine whether the TMTC provided a better care model for MDR-TB patients than the conventional model.

**Methods and Findings:**

A total of 651 pulmonary MDR-TB patients that were diagnosed nation-wide from January 2000-August 2008 were enrolled. Of those, 290 (45%) MDR-TB patients whose initial sputum sample was taken in January 2007 or later were classified as patients in the TMTC era. All others were classified as patients in the pre-TMTC era. The treatment success rate at 36 months was better in the TMTC era group (82%) than in the pre-TMTC era group (61%) (p<0.001). With multiple logistic regressions, diagnosis in the TMTC era (adjusted odds ratio (aOR) 2.8, 95% confidence interval (CI) 1.9–4.2) was an independent predictor of a higher treatment success rate at 36 months. With the time-dependent proportional hazards method, a higher treatment success rate was still observed in the TMTC era group compared to the pre-TMTC era group (adjusted hazard ratio 6.3, 95% CI 4.2–9.5).

**Conclusion:**

The improved treatment success observed in the TMTC era compared to the pre-TMTC era is encouraging. The detailed TMTC components that contribute the most to the improved outcome will need confirmation in follow-up studies with large numbers of MDR-TB patients.

## Introduction

The threat of multidrug-resistant tuberculosis (MDR-TB) to global public health is an important issue. According to the fourth Global Drug Resistance Surveillance Project, 0% to 22.3% of patients had primary multiple drug resistance and 0% to 62.5% had secondary multiple drug resistance [1]. In Taiwan, 1% of new TB cases are diagnosed as MDR tuberculosis, and the prevalence of human immunodeficiency virus is lower than 1% among MDR-TB patients. The total number of MDR-TB patients under case management was approximately 400 to 430 in 2007 [2].

The treatment outcomes of MDR-TB patients were generally worse than patients with drug-sensitive TB, with higher default rates and lower success rates [3]. The World Health Organization (WHO) therefore advocated the strategy of ‘directly observed treatment, short course-plus’ (DOTS-Plus) in 1999. The DOTS-Plus strategy was incorporated into the projects of the Stop TB Partnership established in 2000, and a subgroup called the Green Light Committee was placed in charge [4]. In Latvia, among a cohort of 204 MDR-TB patients with individualized treatment reported in 2000, 66% were cured, 7% died, 13% were lost to follow-up, and 14% failed treatment [5]. In Peru, a DOTS-Plus program operated by non-governmental organizations from the United States reported a cure rate of 66.3% among more than 400 patients with MDR-TB in 2008 [6].

In Taiwan, 299 patients with newly diagnosed pulmonary MDR-TB in a referral center between 1992 and 1996 were followed-up [7]. After six years, 51.2% were cured, 10.4% failed treatment, 9.4% died, and 29.1% defaulted. The high default rate revealed problems with transmission and programmatic failure in the control of MDR-TB. Since 1997, patients with TB have been diagnosed and have received medical care in clinics and hospitals, and the health care facilities have been reimbursed by the National Health Insurance if notification processes were completed by those facilities [8]. The collaboration between public health officials and hospitals was bridged by a National Health Insurance quality assurance program [9]. The DOTS program provided to TB patients by public health sectors beginning in 2006 has also significantly improved the success rate [10]. However, MDR-TB patients who required an injection of aminoglycosides had to visit either prescribed hospitals or a contracted out-patient clinic as often as the prescription was required [11]. Public health nurses were not familiar with the complicated regimens for MDR-TB or the adverse effects related to these drugs compared to those for drug-sensitive TB [11]. Although second-line anti-TB medications were frequently prescribed for twice daily use, only one DOT visit could be provided due to resource limitations and inflexibility of working hours [10]. Although lower adherence to and completion rates of MDR-TB treatment were anticipated due to prolonged treatment and a higher rate of adverse drug effects, an equal amount of incentives and enablers were provided to patients with either drug-sensitive TB or MDR-TB in the DOTS program [11].

In 2006, standardized definitions and treatment guidelines were established for MDR-TB case management that allow comparison between treatment groups and facilitate the development of a more evidence-based approach to different intervention strategies [12], [13]. In May 2007, the Centers for Disease Control, Taiwan (TCDC) launched a new diagnostic and treatment program for MDR-TB according to the guidelines [4],[12], named Taiwan MDR-TB Consortiums (TMTC) [11]. Our aim was to assess whether this patient-centered treatment program, TMTC, could improve the treatment outcome for MDR-TB patients.

## Materials and Methods

### Ethics statement

This project was reviewed by the TCDC, and approved as public health surveillance, which is exempt from human subjects review and does not require informed consent.

### How TMTC provided MDR-TB care

Since 2007, the TMTC has provided general medical care to MDR-TB patients and operated DOTS-Plus projects through five professional therapeutic teams. In contrast to the conventional model of hospital treatment and government-DOT, a hospital-initiated, patient-centered treatment program was begun. Admission to hospitals in the initial scaling-up period for second-line anti-TB medications was not mandatory but encouraged to improve the accommodations for patients experiencing adverse effects due to the medications and to improve the relationships between patients and the team. Designated observers and nurses were employed by therapeutic teams to deliver the correct drugs to patients, typically at their home, including injectable aminoglycosides, as frequently as the patient required. The DOTS-Plus team reported adverse effects or other medical conditions to the physicians who were in direct charge of the program. When patients came to out-patient clinics for refills or check-ups, they were accompanied by team members from the TMTC to address hospital affairs and have examinations done in regards of infection control. Incentives and enablers could be provided directly to the patients as needed with more flexibility compared to government-DOT. Therefore, the DOTS-Plus team in the TMTC not only connected the gaps between physician care and public health control but also facilitated communication and rapport with patients. The regular discussion of difficult cases, regimen consensus, treatment duration, updated diagnostic tools or decisions for treatment failure among the five professional therapeutic teams and experts was organized in quarterly reviews by the TCDC.

### Study population and data collection

**Figure 1:**
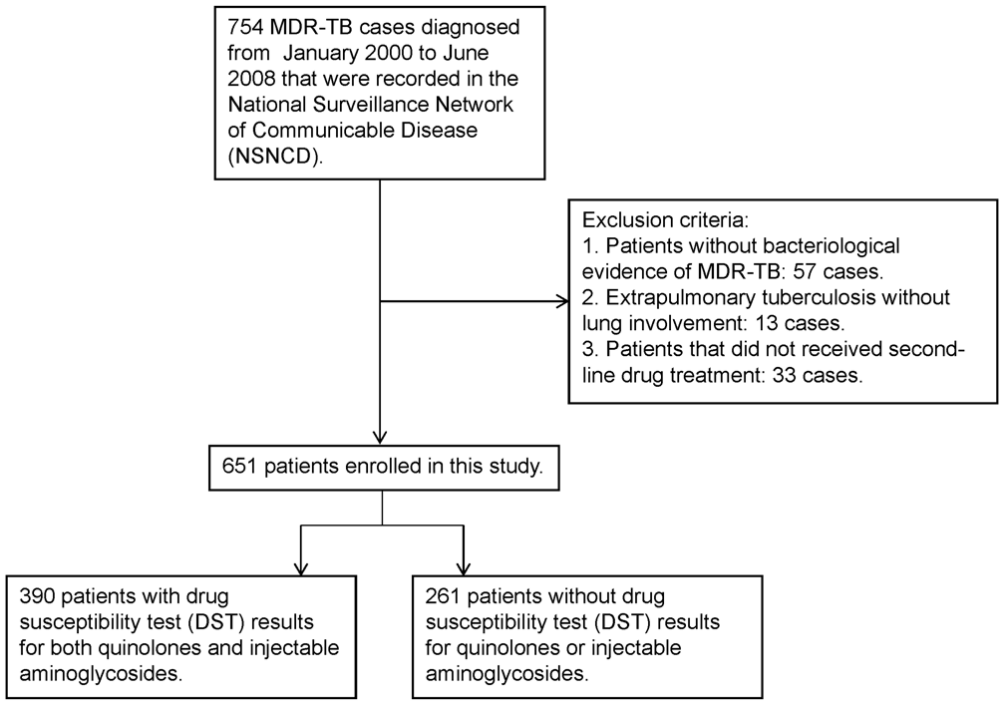
Patient selection. NSNCD, National Surveillance Network of Communicable Disease. MDR, multiple-drug resistance; TB, tuberculosis.

All pulmonary MDR-TB cases that were diagnosed nation-wide between January 2000 and June 2008 were enrolled in the study (Figure 1). MDR-TB patients with positive culture results after January 2007 were informed and consented to participate in the TMTC. The diagnosis, baseline demographics, risk factors, treatment course, and treatment outcomes of patients participating in the TMTC were mandatorily recorded in the TMTC database. As a part of the National TB Program (NTP) evaluation, the effectiveness of TMTC in the field was evaluated. Therefore, MDR-TB patients whose initial sputum was collected in January 2007 or after were defined as patients in the TMTC era. All others were defined as patients in the pre-TMTC era. Information on patients who did not receive TMTC care was collected from chart review, case management cards and the National Surveillance Network of Communicable Disease, TCDC, using a form with the same requirements as the TMTC database [14]. For medical records lacking body weight (3 in the pre-TMTC group) and body height (45 in the pre-TMTC group 11 in the TMTC era), the average body weight and body height were used for the missing data. For incomplete drug susceptibility test (DST) records for first-line medications (both ethambutol and streptomycin in 15, only ethambutol in 4, only streptomycin in 1), the missing data were marked as sensitive.

### Classifications and severity of MDR-TB patients

Previous anti-TB treatment has a strong influence on the prognosis of MDR-TB cases, and registering patients based on their history of anti-TB treatment was therefore important. According to the guidelines released by the WHO, the patients could be classified into six groups [4]. In our analysis, patient classifications were determined by a history of previous treatment recorded at the time of collection of the sputum sample that was later used to confirm the MDR-TB diagnosis. Table S1 shows the median duration and severity of illness at the time of diagnosis among the various categories of treatment history. A preliminary analysis indicated that patients with certain classifications had a similar rate of successful outcomes. Patient classifications were therefore grouped into three categories: new; relapse/treatment after failure of the first treatment/treatment after default; and treatment after failure of re-treatment. As to the severity of patients, three surrogate indicators, “culture converted before using second-line drug”, “smear negative at the time of MDRTB diagnosis” and “cavitary lesion on CXR”, revealed that patients in the TMTC era were diagnosed earlier than patients in the pre-TMTC era. Therefore, they were adjusted or stratified in the following analysis.

### Follow-up and main outcome measure

The treatment outcomes were categorized as cured, treatment completed, died, failed, and defaulted, as defined by the WHO [12]. At the end of follow-up, patients with definitive treatment outcomes, including treatment outcomes after default, were grouped into “successfully treated” (cured and treatment completed) and “not successfully treated”. Any outcome other than cured and treatment completed was grouped into “not successfully treated”. All patients were followed up until they had an outcome result for MDR-TB treatment or until May 31, 2012. For patients “not successfully treated”, the date of failure diagnosis, the mortality date by death certification or the date of default from the program without subsequent available outcomes were used for the date of the end of follow-up.

### Statistical analysis

We assessed treatment effectiveness and good prognostic factors associated with treatment success. For the univariate analysis, we calculated the maximum likelihood estimates and their exact 95% confidence intervals (CIs). We calculated *p*-values using the Mantel-Haenszel chi-squared method or Fisher's exact test. For all statistical tests, we regarded a p value <0.05 as statistically significant. All statistical tests were two sided. The differences in 36-month treatment outcomes were compared with covariates that were adjusted by multiple logistic regressions. The Kaplan-Meier analyses of the sputum conversion and treatment outcomes were compared between patients in the pre-TMTC and TMTC eras with the log-rank test.

Considering the information that was not lost due to the longer follow-up time of patients in the pre-TMTC era, a Cox's proportional hazards model was used to estimate the hazard ratios and the 95% CIs for demographic/clinical characteristics and prognostic factors associated with treatment outcome. Patients were censored from the analysis when they reached their treatment outcome or the end of the study period. After identifying a candidate final Cox's proportional hazards model with an Akaike information criterion (AIC) stepwise variable selection procedure, we tested the required proportional hazards assumption. There are two solutions to handle the violation of the proportional hazards assumption: adding a time-dependent covariate *Z*(*t*) to the Cox's proportional hazards model or fitting a stratified Cox's proportional hazards model. We took the first approach to show the change of the hazard ratio in a covariate over time. Technically, the time-dependent covariate *Z*(*t*) added to the Cox's proportional hazards model was defined as *Z*(*t*) = *Z*×*f*(*t*), where *Z* is the covariate violating the proportional hazards assumption and *f*(*t*) is a chosen monotonic function of survival time *t* (i.e., time to treatment success), such as *t^q^* (*q*≥1) or log(*t*). *q* was determined by locating the value of the power for survival time *t* such that the test of the proportional hazards assumption yielded a non-significant result.

Because resistance to quinolones and injectable aminoglycosides (extensively drug-resistant TB, XDR-TB) has been reported to be associated with poor treatment outcome in patients [15], we further performed a subgroup analysis of patients with DST results for both quinolones and injectable aminoglycosides (Figure 1). Because only 390 (60%) of the enrolled patients had DST results for both quinolones and injectable aminoglycosides, the fitted value of the characteristics generated from the logistic regression analysis of the patient subgroup were weighted and extrapolated back to the entire enrolled population. The weighted multiple logistic analysis and time-dependent survival analysis were fitted using the *R* 2.13.0 software package (R Foundation for Statistical Computing, Vienna, Austria). Other analyses were conducted using SAS software, version 9.2 (SAS Institute, Cary, North Carolina, USA).

## Results

Among the 651 patients enrolled in this study, 290 (45%) were classified as patients in the TMTC era. The median age was 49 (interquartile range: 39-61) years with a male to female ratio of 3.1. Half of the patients (45%) had been tested for HIV; 6 were HIV positive. Overall, the prevalence of HIV was 0.9% (6/651) after confirming with the HIV notification system (TCDC). Additionally, 16% of the patients had hypertension, 36% suffered from diabetes, 9% were hepatitis C carriers, and 8% were hepatitis B carriers. Table 1 shows the characteristics of the patients in the two eras. Table 2 shows that the baseline DSTs for the first-line drugs was compatible between the patients in the two eras.

**Table 1:** Characteristics of the 651 patients.

Characteristics	Total a	TMTC era a	Pre-TMTC era a	p value b
Total	651	290	361	
Male	494 (76)	220 (76)	274 (76)	0.991
BMI				0.716
<22	397 (61)	179 (62)	218 (60)	
22∼26	191 (29)	86 (30)	105 (29)	
>26	63 (10)	25 (9)	38 (11)	
Age				0.283
<35	128 (20)	58 (20)	70 (19)	
35∼60	346 (53)	145 (50)	201 (56)	
>60	177 (27)	87 (30)	90 (25)	
Risk factors				
Aboriginal	124 (19)	46 (16)	78 (22)	0.064
Alcohol	125 (19)	42 (14)	83 (23)	0.006
Diabetics	234 (36)	82 (28)	152 (42)	<0.001
Hypertension	104 (16)	51 (18)	53 (15)	0.315
Hepatitis B	51 (8)	19 (7)	32 (9)	0.275
Hepatitis C	56 (9)	26 (9)	30 (8)	0.767
Disease severity and delayed diagnosis				
Cavitary lesion on CXR	282 (43)	108 (37)	174 (48)	0.005
Sputum				
Smear-negative at the time of MDR-TB diagnosis	232 (36)	120 (41)	112 (31)	0.006
Culture converted before using second-line drug	145 (22)	81 (28)	64 (18)	0.002
Number of first-line drugs to which isolate is resistant (≥3)	308 (47)	136 (47)	172 (48)	0.849
No Treatment delay c	457 (70)	235 (81)	222 (61)	<0.001
Patient classification				<0.001
New	245 (38)	118 (41)	127 (35)	
Relapse	171 (26)	91 (31)	80 (22)	
Treatment after default	57 (9)	13 (4)	44 (12)	
Treatment after failure of the first treatment	122 (19)	56 (19)	65 (18)	
Treatment after failure of re-treatment	56 (9)	12 (4)	44 (12)	

a Data summarized as n (%).

b Chi-squared test.

c Treatment delay: the lag between sputum collection of MDR-TB and start of second-line drug >120 days.

Abbreviations: BMI: body mass index; CXR: chest X-ray; MDR: multidrug-resistant; TB: tuberculosis; TMTC: Taiwan Multi-drug Resistance Tuberculosis Consortiums.

**Table 2:** Available DST results, drug resistance pattern and regimens prescribed in the beginning of the treatment of MDR-TB.

	DST performed for drug at the time when MDR-TB diagnosed (N = 651)		Patients with resistance to drug detected out of DST performed		Patients treated b (N = 651)	
Drug	TMTC era a	Pre-TMTC era a	TMTC era a	Pre-TMTC era a	TMTC era a	Pre-TMTC era a
**First-line drugs**						
Rifampin	290(100)	361(100)	290(100)	361(100)	55(19)	78(22)
Isoniazid	290(100)	361(100)	290(100)	361(100)	112(39)	106(29)
Ethambutol	273(94)	354(98)	74(27)	96(27)	218(75)	255(71)
Pyrazinamide	11(4)	11(4)	4(36)	9(43)	212(73)	240(66)
Streptomycin	276(95)	355(98)	101(37)	113(32)	108(37)	173(48)
**Second-line drugs**						
**Aminoglycosides**						
Kanamycin	154(53)	138(38)	10(6)	11(8)	114(39)	65(18)
Amikacin	5(2)	2(1)	1(20)	0(0)	2(1)	7(2)
**Other injectables**						
Capreomycin	90(31)	4(1)	3(3)	0(0)	0(0)	1(0.3)
**Fluoroquinolones**						
Ciprofloxacin	0(0)	0(0)	—	—	2(1)	9(2)
Ofloxacin	108(37)	95(26)	24(22)	24(25)	2(1)	33(9)
Levofloxacin	79(27)	40(11)	14(18)	8(20)	90(31)	212(59)
Moxifloxacin	49(17)	36(10)	8(16)	7(19)	171(59)	68(19)
**Thioanamides**						
Prothionamide	141(49)	114(32)	17(12)	23(20)	194(67)	245(68)
**Other second-line agents**						
Cycloserine	4(1)	0(0)	0(0)	0(0)	120(41)	27(7)
Para-aminosalicylic acid	156(54)	126(35)	13(8)	22(17)	95(33)	199(55)
Rifabutin	119(41)	11(3)	98(82)	7(64)	4(1)	13(4)
Terizidone	—	—	—	—	0(0)	0(0)
Linezolid	—	—	—	—	1(0.3)	0(0)
**Third-line drugs**						
Amoxicillin	—	—	—	—	1(0.3)	1(0.3)
Clofazimine	—	—	—	—	0(0)	2(1)
Clarithromycin	—	—	—	—	0(0)	0(0)

a Data summarized as n (%) .

b Regimens prescribed in the beginning of the treatment of MDR-TB.

Abbreviations: DST: drug susceptibility test; MDR: multidrug-resistant; TB: tuberculosis; TMTC: Taiwan Multi-drug Resistance Tuberculosis Consortiums.

### Crude comparison rate

**Figure 2:**
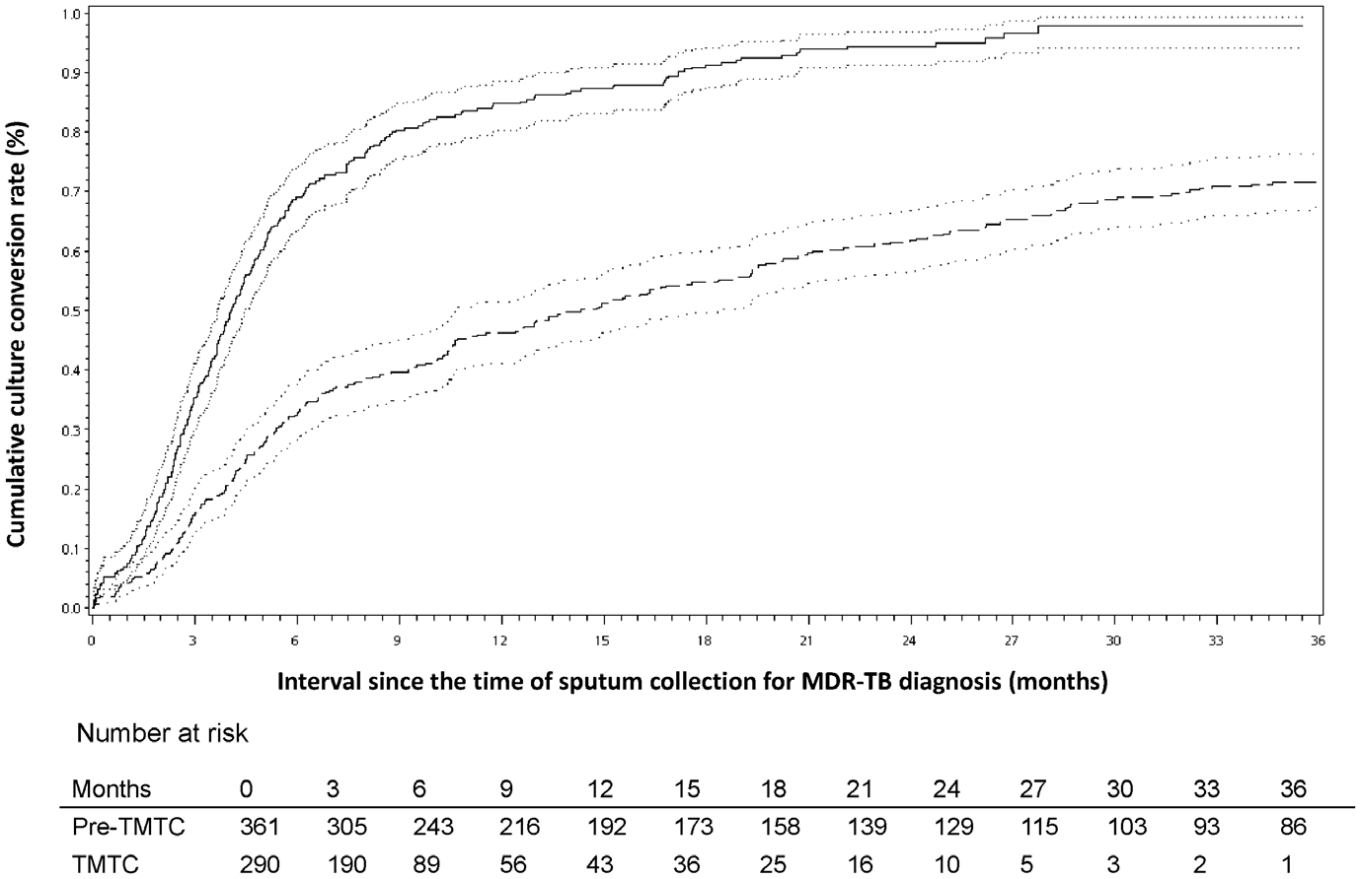
A comparison of the cumulative sputum conversion rates of multidrug-resistant tuberculosis (MDR-TB) cases in the pre-Taiwan MDR-TB Consortium (TMTC) era and the TMTC era. Dashed line, pre-TMTC era; solid line, TMTC era; dotted line, 95% confidence interval (p<0.001 by the log-rank test).

**Figure 3:**
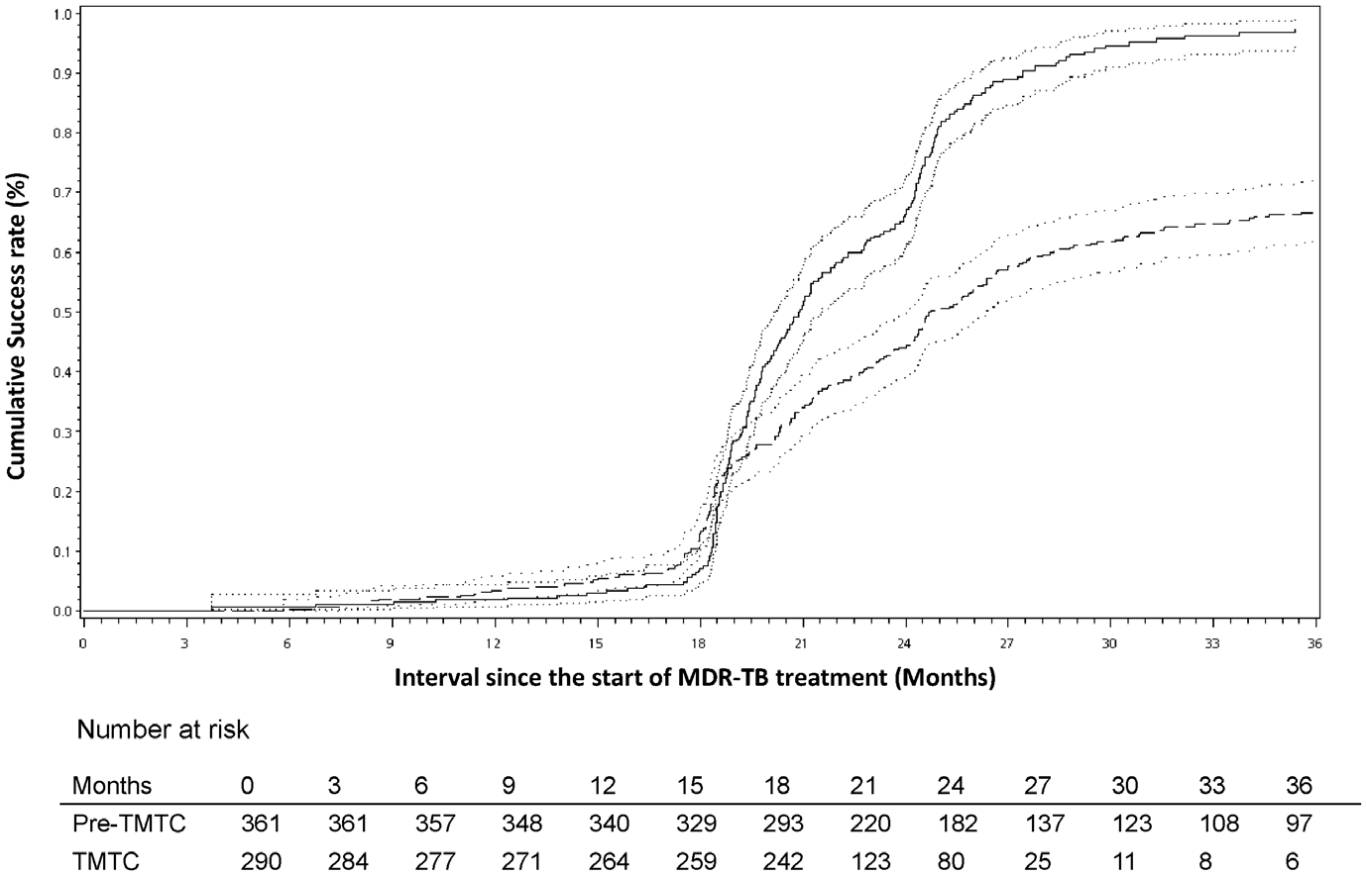
Treatment outcomes of 651 multidrug-resistant tuberculosis (MDR-TB) patients after the administration of second-line drugs. The cumulative success rate reveals that patients in the Taiwan MDR-TB Consortium (TMTC) era had a higher probability of treatment success than patients in the pre-TMTC era. Dashed line, pre-TMTC era; solid line, TMTC era; dotted line, 95% confidence interval (*p*<0.001 by the log-rank test).

At 6, 12 and 18 months after sputum was collected for the diagnosis of MDR-TB, the conversion rates were 33%, 46% and 54%, respectively, in the pre-TMTC era vs. 68%, 83%, and 90%, respectively, in the TMTC era (chi-squared, p<0.001). The Kaplan-Meier analysis of sputum conversion revealed that a 50% conversion occurred at 14.3 (95% confidence interval [CI] 10.6–18.3) months in the pre-TMTC era, whereas it occurred at 4.0 (95% CI 3.7–4.4) months in the TMTC era (log-rank test, *P*<0.001) (Figure 2). If we calculated the median time for conversion from the 457 patients who remained culture positive when they started second-line medications, the median culture conversion time (calculated from the start day of the second-line medications) was only 2 (95% CI 1.7–2.5) months in the TMTC era and 6.6 (95% CI 4.5–9.7) months in the pre-TMTC era. At 24 and 36 months after the administration of second-line anti-TB drugs, the success rates were 24% and 44%, respectively, in the pre-TMTC era vs. 59% and 81%, respectively, in the TMTC era (chi-squared, p<0.001, respectively). The Kaplan-Meier analysis of treatment success revealed that 50% success occurred at 24.7 (95% confidence interval (CI) 24.0–26.4) months after the administration of second-line drugs in the pre-TMTC era, whereas it occurred at 20.9 (95% CI 20.1–21.5) months in the TMTC era (log-rank test, p<0.001) (Figure 3). The median duration of treatment in the pre-TMTC era was 25.5 (2.8–90.4) months, which was significantly longer than the 20.7 (1.0–47.9) months observed in the TMTC era (Wilcoxon two-sample test, p<0.001).

### Prognostic factors for treatment success

Table 3 shows the characteristics of the 651 patients with treatment success at 36 months after the administration of second-line drugs. Patients who were diagnosed in the TMTC era were 2.8-fold (95% CI 1.9–4.2, p<0.001) more likely to have treatment success at 36 months compared to patients in the pre-TMTC era, after covariate adjustment. Table 4 lists the significant prognostic factors associated with treatment success in the time-dependent Cox's regression model with an adequate fit (adjusted generalized *R* ^2^ = 0.21 > 0.15). In this regression model, TMTC×*t* ^1.88^ was the sole time-dependent covariate for satisfying the proportional hazards assumption, where TMTC = 1 for the TMTC era and 0 for the pre-TMTC era, *t* was the time to success (in days), and q = 1.88 was determined by locating the value of the power for survival time *t* such that the test of proportional hazards assumption yielded a non-significant result. A higher treatment success rate was still observed in the TMTC era group compared to the pre-TMTC era group (adjusted hazard ratio 6.3, 95% CI 4.2–9.5, p<0.001).

**Table 3:** Prognostic factors associated with treatment success.

Covariate	Classification (n)	n (%) of treatment success	Univariable OR (95% CI)	p value	Multivariate aOR a (95% CI)	p value
Group	TMTC era (290)	239 (82)	2.9(2.0–4.2)	<0.001	2.8(1.9–4.2)	<0.001
	Pre-TMTC era (361)	222 (61)	Reference		Reference	
Sex	Male (494)	341 (69)	0.7(0.5–1.0)	0.076	0.8(0.5–1.3)	0.351
	Female (157)	120 (76)	Reference		Reference	
Aboriginal	Yes (124)	88 (71)	1.0(0.7–1.6)	0.967	—	
	No (527)	373 (71)	Reference		—	
BMI	<22 (397)	270 (68)	Reference		Reference	
	22∼26 (191)	146 (76)	1.5(1.0–2.3)	0.036	1.7(1.1–2.6)	0.021
	>26 (63)	45 (71)	1.2(0.7–2.1)	0.588	1.1(0.6–2.2)	0.709
Age	<35 (128)	102 (80)	2.3(1.4–4.0)	0.002	2.6(1.4–4.8)	0.001
	35∼60 (346)	248 (72)	1.5(1.0–2.2)	0.037	2.1(1.4–3.3)	<0.001
	>60 (177)	111 (63)	Reference		Reference	
Alcohol	Yes (125)	82 (66)	0.7(0.5–1.1)	0.155	—	
	No (526)	379 (72)	Reference		—	
Diabetics	Yes (234)	157 (67)	0.8(0.5–1.1)	0.118	—	
	No (417)	304 (73)	Reference		—	
Hypertension	Yes (104)	72 (69)	0.9(0.6–1.4)	0.698	—	
	No (547)	389 (71)	Reference		—	
Hepatitis B	Yes (51)	36 (71)	1.0(0.5–1.9)	0.971	—	
	No (600)	425 (71)	Reference		—	
Hepatitis C	Yes (56)	29 (52)	0.4(0.2–0.7)	0.001	0.4(0.2–0.7)	0.002
	No (595)	432 (73)	Reference		Reference	
Cavitary lesion on CXR	Yes (282)	182 (65)	0.6(0.4–0.8)	0.002	0.6(0.4–0.9)	0.023
	No (369)	279 (76)	Reference		Reference	
Smear-negative at the time of MDR-TB diagnosis	Negative (232)	178 (77)	1.6(1.1–2.3)	0.014	—	
	Positive (419)	283 (68)	Reference		—	
Culture converted before second-line drug	Negative (145)	117 (81)	2.0(1.3–3.1)	0.003	1.5(0.9–2.5)	0.100
	Positive (506)	344 (68)	Reference		Reference	
Number of first-line drugs to which isolate is resistant	≥3 (308)	201 (65)	0.6(0.4–0.8)	0.003	0.6(0.4–0.8)	0.002
	<3 (343)	260 (76)	Reference		Reference	
Treatment delay b	No (457)	328 (72)	1.2(0.8–1.7)	0.409	—	
	Yes (194)	133 (69)	Reference		—	
Patient classification	New (245)	194 (79)	8.7(4.6–16.7)	<0.001	7.0(3.5–14.0)	<0.001
	Relapse+ treatment after default+ treatment after failure of the first treatment (350)	250 (71)	5.8(3.1–10.6)	<0.001	5.1(2.7–9.9)	<0.001
	Treatment after failure of re-treatment (56)	17 (30)	Reference		Reference	

a Multiple logistic analysis, adjusted with covariates chosen by Akaike information criterion (AIC).

b Treatment delay: the lag between sputum collection of MDR-TB and start of second-line drug > 120 days.

Abbreviations: aOR: adjusted odds ratio; BMI: body mass index; CI: confidence interval; CXR: chest X-ray; MDR: multidrug-resistant; TB: tuberculosis; TMTC: Taiwan Multi-drug Resistance Tuberculosis Consortiums.

**Table 4:** Prognostic factors associated with treatment success in a time-dependent Cox regression model.

Covariate	Classification (n)	Multivariate a aHR (95% CI)	p value	Multivariate aHR b (95% CI)	p value
TMTC era	24 months (290)	2.3 (1.9–2.8)	<0.001	2.4 (1.9–2.9)	<0.001
	36 months (290)	6.0 (3.9–9.1)	<0.001	6.3 (4.2–9.5)	<0.001
Sex	Male (494)	0.9 (0.8–1.2)	0.579	0.9 (0.8–1.1)	0.477
	Female (157)	Reference		Reference	
Aboriginal	Yes (124)	1.2 (1.0–1.6)	0.080	1.2 (1.0–1.5)	0.092
	No (527)	Reference		Reference	
BMI	<22 (397)	Reference		—	
	22∼26 (191)	1.0 (0.8–1.2)	0.989	—	
	>26 (63)	1.2 (0.9–1.7)	0.206	—	
Age	<35 (128)	0.9 (0.7–1.2)	0.334	0.9 (0.7–1.1)	0.237
	35∼60 (346)	0.8 (0.6–1.0)	0.083	0.8 (0.6–1.0)	0.021
	>60 (177)	Reference		Reference	
Alcohol	Yes (125)	0.9 (0.7–1.1)	0.368	—	
	No (526)	Reference		—	
Diabetics	Yes (234)	1.0 (0.8–1.2)	0.664	—	
	No (417)	Reference		—	
Hypertension	Yes (104)	1.1 (0.9–1.4)	0.375	—	
	No (547)	Reference		—	
Hepatitis B	Yes (51)	1.0 (0.7–1.4)	0.863	—	
	No (600)	Reference		—	
Hepatitis C	Yes (56)	0.7 (0.5–0.9)	0.022	0.7 (0.5–1.0)	0.030
	No (595)	Reference		Reference	
Cavitary lesion on CXR	Yes (282)	0.9 (0.7–1.1)	0.240	—	
	No (369)	Reference		—	
Smear-negative at the time of MDR-TB diagnosis	Negative (232)	1.1 (0.9–1.3)	0.378	—	
	Positive (419)	Reference		—	
Number of first-line drugs to which isolate is resistant	≥3 (308)	0.9 (0.8–1.1)	0.322	—	
	<3 (343)	Reference		—	
Treatment delay c	No (457)	0.8 (0.6–0.9)	0.012	0.8 (0.6–1.0)	0.018
	Yes (194)	Reference		Reference	
Patient classification	New (245)	3.5 (2.3–5.1)	<0.001	3.6 (2.5–5.4)	<0.001
	Relapse+ treatment after default+ treatment after failure of the first treatment (350)	2.5 (1.7–3.6)	<0.001	2.5 (1.7–3.7)	<0.001
	Treatment after failure of re-treatment (56)	Reference		Reference	

a Adjusted with all covariates and stratified with culture converted before second-line drug.

b Adjusted with covariates chosen by Akaike information criterion (AIC) and stratified with culture converted before second-line drug.

c Treatment delay: the lag between sputum collection of MDR-TB and start of second-line drug > 120 days.

Abbreviations: aHR: adjusted hazard ratio; BMI: body mass index; CI: confidence interval; CXR: chest X-ray; TMTC: Taiwan Multi-drug Resistance Tuberculosis Consortiums.

### Subgroup analysis

A subgroup of 390 patients (60%) had DST results for both quinolones and injectable aminoglycosides. Table 5 shows the characteristics of 390 patients with treatment success at 36 months after the administration of second-line drugs and the result of the unadjusted logistic regression analysis. Patients who were diagnosed in the TMTC era were still more likely to have treatment success at 36 months compared to patients in the pre-TMTC era (adjusted odds ratio (aOR) 5.5, 95% CI 2.5–8.7, p<0.001) after covariate adjustment. Patients with DST resistance to quinolones only or injectable aminoglycosides only were more likely to have treatment success compared to patients with XDR-TB. Patients with DST resistance to quinolones only or who had XDR-TB were 70% and 90% less likely to have treatment success, respectively, compared to patients without resistance to both drugs (aOR 0.4, 95% CI 0.2–0.6, p<0.001; aOR 0.1, 95% CI 0.1–0.3, p<0.001), whereas patients with DST resistance to injectable aminoglycosides only did not demonstrate different treatment success rates (aOR 1.5, 95% CI 0.4–5.8, p = 0.584) compared to patients without resistance to both drugs.

**Table 5:** Prognostic factors associated with treatment success for 390 patients with results of susceptibility of second-line drug.

Covariate	Classification (n)	n (%) of treatment success	Univariable OR (95% CI)	p value	Multivariate aOR a (95% CI)	p value
Group	TMTC era (200)	161 (81)	5.3 (3.4–8.4)	<0.001	5.5 (3.5–8.7)	<0.001
	Pre-TMTC era (190)	83 (44)	Reference		Reference	
Sex	Male (296)	177 (60)	0.6 (0.4–1.0)	0.046	0.8 (0.5–1.4)	0.600
	Female (94)	67 (71)	Reference		Reference	
Aboriginal	Yes (70)	39 (56)	0.7 (0.4–1.2)	0.192	0.6 (0.4–1.1)	0.058
	No (320)	205 (64)	Reference		Reference	
BMI	<22 (239)	144 (60)	Reference		Reference	
	22∼26 (109)	71 (65)	1.2 (0.8–2.0)	0.385	1.5 (0.9–2.4)	0.138
	>26 (42)	29 (69)	1.5 (0.7–3.0)	0.282	1.8 (0.9–3.5)	0.042
Age	<35 (81)	59 (73)	2.2 (1.1–4.0)	0.018	4.7 (2.4–9.1)	<0.001
	35∼60 (210)	130 (62)	1.3 (0.8–2.1)	0.289	4.0 (2.4–6.7)	<0.001
	>60 (99)	55 (56)	Reference		Reference	
Alcohol	Yes (65)	30 (46)	0.4 (0.3–0.8)	0.003	0.4 (0.2–0.6)	<0.001
	No (325)	214 (66)	Reference		Reference	
Diabetics	Yes (140)	78 (56)	0.6 (0.4–1.0)	0.037	—	
	No (250)	166 (66)	Reference		—	
Hypertension	Yes (60)	36 (60)	0.9 (0.5–1.5)	0.656	—	
	No (330)	208 (63)	Reference		—	
Hepatitis B	Yes (31)	18 (58)	0.8 (0.4–1.7)	0.590	—	
	No (359)	226 (63)	Reference		—	
Hepatitis C	Yes (40)	18 (45)	0.5 (0.2–0.9)	0.017	0.3 (0.2–0.6)	0.003
	No (350)	226 (65)	Reference		Reference	
Cavitary lesion on CXR	Yes (181)	101 (56)	0.6 (0.4–0.9)	0.010	0.5 (0.3–0.8)	0.008
	No (209)	143 (68)	Reference		Reference	
Smear-negative at the time of MDR-TB diagnosis	Negative (123)	85 (69)	1.5 (1.0–2.4)	0.071	—	
	Positive (267)	159 (60)	Reference		—	
Culture converted before second-line drug	Negative (59)	44 (75)	1.9 (1.0–3.6)	0.041	—	
	Positive (331)	200 (60)	Reference		—	
Number of first-line drugs to which isolate is resistant	≥3 (189)	106 (56)	0.6 (0.4–0.9)	0.011	0.5 (0.3–0.7)	<0.001
	<3 (201)	138 (69)	Reference		Reference	
Treatment delay b	No (273)	170 (62)	1.0 (0.6–1.5)	0.855	0.6 (0.4–0.9)	0.010
	Yes (117)	74 (63)	Reference		Reference	
Drug susceptibility test	Quinolone resist (87)	36 (41)	2.1 (0.9–4.6)	0.081	2.9 (1.4–6.1)	0.002
	Injection resist (10)	8 (80)	11.6 (2.1–63.3)	0.005	11.7 (2.6–51.8)	<0.001
	No resist (250)	189 (76)	9.0 (4.3–19.0)	<0.001	7.9 (3.9–16.0)	<0.001
	Both resist (43)	11 (26)	Reference		Reference	
Patient classification	New (128)	96 (75)	9.0 (4.2–19.4)	<0.001	4.0 (1.8–8.9)	<0.001
	Relapse+ treatment after default + treatment after failure of the first treatment (214)	136 (64)	5.2 (2.6–10.6)	<0.001	2.7 (1.2–5.9)	0.011
	Treatment after failure of re-treatment(48)	12 (25)	Reference		Reference	

a Multiple logistic analysis, adjusted with covariates chosen by Akaike information criterion (AIC).

b Treatment delay: the lag between sputum collection of MDR-TB and start of second-line drug > 120 days.

Abbreviations: aOR: adjusted odds ratio; BMI: body mass index; CI: confidence interval; CXR: chest X-ray; MDR: multidrug-resistant; OR: odds ratio; TB: tuberculosis; TMTC: Taiwan Multi-drug Resistance Tuberculosis Consortiums.

## Discussion

In this study, after adjusting for patient classification and other important covariates, treatment success was significantly improved among patients in the TMTC era compared to the pre-TMTC era in several different models. The better treatment results at 36 months and the long-term follow-up highlighted the effectiveness of early intervention and a patient-centered DOTS-Plus project, irrespective of patient classifications of MDR-TB cases. The TMTC, with the objectives of combating the high default rate and the emerging number of MDR-TB cases by integrating medical resources and the DOTS-Plus project [11], improved the cure rate and decreased the incidence of default.

It is possible that the subgroup analysis of the 390 patients with available DSTs for second-line drugs did not represent the 651 patients included in the original cohort. However, a comparison between those with or without a DST for second-line drugs revealed that those with DSTs were typically cases with higher severity (Table S2). Even so, the analysis in table 5 showed the same trend for TMTC effectiveness as the result in table 3. This further suggests that TMTC was useful for advanced MDR-TB patients.

The duration of treatment was significantly longer in the pre-TMTC era patients (Figure 2). Compared to the community-based MDR-TB management program at the Indus Hospital in Karachi, Pakistan, between January 2008 and June 2010, the median time of culture conversion in Karachi was 196 days, which was longer than the 160 days in the TMTC era but shorter than the 14.3 months in the pre-TMTC era [16]. Because TMTC was implemented in May 2007, some patients who failed to complete treatment before that date were re-evaluated by therapeutic teams and retreated under supervision, leading to a longer duration of treatment. However, the significantly shorter duration of treatment in the TMTC era indicates that a patient-centered treatment program has potential benefits. Nevertheless, the effectiveness of TMTC improved when we excluded 311 patients from the pre-TMTC era who had treatment results in the TMTC era (Table S3). Compared to a study analyzing the treatment outcomes of 1027 patients with MDR-TB in Latvia between 2000 and 2004 [17], the duration of treatment for those categorized as treatment failure was longer in Taiwan than in Latvia (708 days versus 348 days, respectively), which could be the result of physician-dependent judgment to define treatment failure in Taiwan. Although standardized treatment outcome measures for MDR-TB have been proposed to allow international comparisons [13], the definition of treatment failure is still debated [18]. Because ineffective treatment leads to the unnecessary expenditure of medical resources, a consensus for a more sophisticated definition of MDR-TB treatment failure is mandatory.

The best strategy to prevent MDR-TB transmission is to treat MDR-TB cases to prevent chronicity. Experiences in Peru, Russia and Lesotho revealed that the six elements for the successful implementation of MDR-TB programs are as follows: the performance of baseline assessments, the early identification of key collaborators, the identification of an initial locus of care, the minimization of patient-incurred costs, targeted interventions for vulnerable populations, and the importance of technical assistance and funding [19]. Countries in the Asia-Pacific area, such as South Korea and Taiwan, have well-established medical care systems and sound public health infrastructures. However, only 45.3% of treatment successes in all registered MDR-TB patients in 2000–2002 were achieved in South Korea [15], similar to the 44% in all registered MDR-TB patients in the pre-TMTC era (2000–2006) in our study. There was an even worse report from South Korea in 2011, showing that only 37.1% had a successful outcome in a 2004 cohort receiving care in three public referral pulmonary hospitals [20]. No DOT was specifically mentioned, and a high default rate (reaching 37.1%) in MDR-TB patients accompanied by a high proportion of XDR-TB (15%) cases was the cause of the lower rate of successful outcomes. In Shanghai, 53.1% of MDR-TB patients (excluding XDR-TB cases) enrolled from July 2007 to June 2009 in a pulmonary hospital had a successful outcome, which was much lower than the 81% observed in the TMTC era [21]. The lack of a comprehensive TB control program including socioeconomic support, an adequate follow-up system, an infection control program, careful management of comorbidities, and proper case management could have caused the low success and high default rates in this area. Fluoroquinolones and other second-line drugs used without restriction in the private sector for TB patients and those with community-acquired pneumonia, raising the concern of very limited numbers of effective anti-XDR-TB medications in both countries. With a relatively stable public health infrastructure and an easily accessible medical care system [22], the identification of vulnerable populations and the initial locus of care became feasible in Taiwan. With the technical assistance of the NTP and their strong political will, the TMTC incorporated a good-quality private-public mix model to deliver creative care to MDR-TB patients. The TMTC model could be a solution for the NTP in this area to address the poor outcomes of MDR-TB patients and the emerging XDR-TB problem before there are no effective medications to block transmission in this area.

There were many limitations to our study. The TMTC was scaled up very fast in 2007; therefore, the high TMTC coverage of MDR-TB patients prevented us from using contemporary controls. The use of historical controls rather than contemporary controls introduced a temporal bias (e.g., patients treated more recently did better because the treating personnel were more experienced) and other factors (e.g., improved adherence to MDR-TB treatment guidelines, more medications available for MDR-TB treatment) that may have affected the difference in outcomes in a manner not related to the TMTC program. The median number of sputum examinations per month was 1.5 in the TMTC era compared to 1.0 in the pre-TMTC era. Individualized regimens were provided in both groups mainly by the private sector under the NHI. However, the MDR-TB treatment provided to patients in the TMTC era was required to meet the WHO MDR-TB treatment guidelines (Table 2). The improvement of the adherence to standard regimens and the increased frequency of sputum collections during treatment were considered as achievements of the TMTC program. Therefore, we did not use these covariates adjusted in analysis due to potential co-linearity. We also evaluated the evolution of treatment success in MDR-TB patients in Taiwan in the past two decades. Only 47% of 36 MDR-TB patients achieved sustained culture conversion at TB centers in Taipei City during 1987–1989 [23]. The treatment success of MDR-TB patients in 1992-1996 was 51.2% at a referral center in Taipei City [7], while it was 44% in all registered MDR-TB patients in 2000–2006 in this study. Ofloxacin had been utilized for MDR-TB patients in Taiwan since 1992 [24], but the regimens for MDR-TB patients were not always adequate because TB patients could receive TB care in any NHI-contracted hospital. Some effective second-line anti-TB medications were not available for all MDR-TB patients until the TMTC was created. The TMTC itself therefore represented the designed regimens, the quality of the DOT and patient-centered care as the entirety of medical care and case management. Before the TMTC was created, we can conclude that treatment success was consistently poor even with the use of ofloxacin and newer generations of fluoroquinolones. The treatment outcome for MDR-TB patients in 2007–2008 (the TMTC era) achieved 81%. This study enrolled all the MDR-TB patients reported during the study period to prevent selection bias and provided strong evidence that better outcomes were achieved in the TMTC era.

In the TMTC era, each strain of MDR-TB was required to be transferred to the Reference Laboratory of Mycobacteriology, TCDC, where MDR-TB confirmation testing was performed [25]. Rapid diagnostic tools, such as molecular line-probe assays, were not used in the study period. Before 2007, DSTs of second-line drugs were performed by the clinical microbiology laboratories of the hospitals using agar proportional methods only at the request of clinicians [23]. Thus, a difference in the chance of misclassification of the DST results of second-line drugs may exist between the pre-TMTC era and the TMTC era. Moreover, the systemic DST surveillance of second-line drugs in MDR-TB patients was required after 2007. Therefore, 52% of patients in the pre-TMTC era underwent DSTs for second-line drugs, while 69% of those in the TMTC era had DSTs for second-line drugs. However, this difference would not affect the better treatment outcomes in the TMTC era in the analysis of the 651 patients (Tables 3 and 5). We acknowledged some challenges, but we also demonstrated the feasibility of using a weighted logistic analysis to adjust for covariates and demonstrate an overall improvement in MDR-TB case management.

In conclusion, the improved treatment success achieved in the TMTC era compared to the pre-TMTC era is encouraging. The detailed components of the TMTC that contributed the most to the better outcomes require confirmation in follow-up studies with larger numbers of MDR-TB patients.

## Supporting Information

Table S1 **The median duration and severity of illness at the time of diagnosis among the various categories of treatment history.**
(DOCX)

Table S2 **Basic characteristics of patients with or without second-line anti-TB DST.**
(DOCX)

Table S3 **Prognostic factors associated with treatment success for 340 patients (excluding 311 patients in pre-TMTC era with treatment results in TMTC era).**
(DOCX)
