# Supplementary material for: Effectiveness of a Government-Organized and Hospital-Initiated Treatment for Multidrug-Resistant Tuberculosis Patients-A Retrospective Cohort Study
Source: PLoS One. 2013 Feb 25;8(2):e57719. doi: 10.1371/journal.pone.0057719 (PMC3581541; doi:10.1371/journal.pone.0057719)
Supplement: Table S1 — The median duration and severity of illness at the time of diagnosis among the various categories of treatment history. (DOCX) [file pone.0057719.s001.docx]

**Table S1.** The median duration and severity of illness at the time of diagnosis among the various categories of treatment history

| **Characteristics** | **Total ^a^** | **Smear-negative at the time of MDR-TB diagnosis ^a^** | **Culture converted before second-line drug ^a^** | **Cavitary lesion on CXR ^a^** | **Median duration of illness ^b^** |
| --- | --- | --- | --- | --- | --- |
| Total | 651 | 232 (36) | 145(22) | 282 (43) | - |
| New | 245 (38) | 93 (38) | 69 (28) | 84 (34) | -1 (96) |
| Relapse | 171 (26) | 65 (38) | 43 (25) | 76 (44) | 1451(1787) |
| Treatment after default | 57 (9) | 19 (33) | 8 (14) | 32 (56) | 1115(1433) |
| Treatment after failure of the first treatment | 122 (19) | 45 (37) | 18 (15) | 56 (46) | 247(538) |
| Treatment after failure of re-treatment | 56 (9) | 10 (18) | 7 (13) | 34 (61) | 1532(1589) |

^a^Data summarized as n (%) .

^b^The duration between notification as a TB case until the sputum collection for MDR-TB diagnosis; Values are median (mean)

Abbreviations: CXR: chest radiograph; MDR: multidrug-resistant; TB: tuberculosis.
