# Supplementary material for: Effectiveness of a Government-Organized and Hospital-Initiated Treatment for Multidrug-Resistant Tuberculosis Patients-A Retrospective Cohort Study
Source: PLoS One. 2013 Feb 25;8(2):e57719. doi: 10.1371/journal.pone.0057719 (PMC3581541; doi:10.1371/journal.pone.0057719)
Supplement: Table S2 — Basic characteristics of patients with or without second-line anti-TB DST. (DOCX) [file pone.0057719.s002.docx]

**Table S2.** Basic characteristics of patients with or without second-line anti-TB DST.

| **Characteristics** | **Total ^a^** | **Patients with second-line anti-TB DST ^a^** | **Patients without second-line anti-TB DST ^a^** | **p value ^b^** |
| --- | --- | --- | --- | --- |
| Total | 651 | 390 | 261 |  |
| TMTC-era | 290 (45) | 200 (51) | 90 (34) | <0.001 |
| Male | 494 (76) | 296 (76) | 198 (76) | 0.992 |
| BMI |  |  |  | 0.396 |
| <22 | 397 (61) | 239 (61) | 158 (61) |  |
| 22~26 | 191 (29) | 109 (28) | 82 (31) |  |
| >26 | 63 (10) | 42 (11) | 21 (8) |  |
| Age |  |  |  | 0.395 |
| <35 | 128 (20) | 81 (21) | 47 (18) |  |
| 35~60 | 346 (53) | 210 (54) | 136 (52) |  |
| >60 | 177 (27) | 99 (25) | 78 (30) |  |
| Risk factors |  |  |  |  |
| Aboriginal | 124 (19) | 70 (18) | 54 (21) | 0.383 |
| Alcohol | 125 (19) | 65 (17) | 60 (23) | 0.045 |
| Diabetics | 234 (36) | 140 (36) | 94 (36) | 0.975 |
| Hypertension | 104 (16) | 60 (15) | 44 (17) | 0.615 |
| Hepatitis B | 51 (8) | 31 (8) | 20 (8) | 0.894 |
| Hepatitis C | 56 (9) | 40 (10) | 16 (6) | 0.066 |
| Disease severity and delayed diagnosis |  |  |  |  |
| Cavitary lesion on CXR | 282 (43) | 181 (46) | 101 (39) | 0.052 |
| Sputum |  |  |  |  |
| Smear-negative at the time of MDR-TB diagnosis | 232 (36) | 123 (32) | 109 (42) | 0.008 |
| Culture converted before using second-line drug | 145 (22) | 59 (15) | 86 (33) | <0.001 |
| Number of first-line drugs to  which isolate is resistant |  |  |  | 0.473 |
| ≧3 | 308 (47) | 189 (48) | 119 (46) |  |
| ^c^No Treatment delay | 457 (70) | 273 (70) | 184 (70) | 0.892 |
| Patient classification |  |  |  | <0.001 |
| New | 245 (38) | 128 (33) | 117 (45) |  |
| Relapse | 171 (26) | 114 (29) | 57 (22) |  |
| Treatment after default | 57 (9) | 25 (6) | 32 (12) |  |
| Treatment after failure of the first treatment | 122 (19) | 75 (19) | 47 (18) |  |
| Treatment after failure of re-treatment | 56 (9) | 48 (12) | 8 (3) |  |

^a^Data summarized as n (%) .

^b^Chi-square test.

^c^Treatment delay: the lag between sputum collection of MDR-TB and start of second-line drug > 120 days.

Abbreviations: BMI: body mass index; CXR: chest radiograph; DST: drug susceptibility test; MDR: multidrug-resistant; TB: tuberculosis; TMTC: Taiwan Multi-drug Resistance Tuberculosis Consortiums.
