# Supplementary material for: Effectiveness of a Government-Organized and Hospital-Initiated Treatment for Multidrug-Resistant Tuberculosis Patients-A Retrospective Cohort Study
Source: PLoS One. 2013 Feb 25;8(2):e57719. doi: 10.1371/journal.pone.0057719 (PMC3581541; doi:10.1371/journal.pone.0057719)
Supplement: Table S3 — Prognostic factors associated with treatment success for 340 patients (excluding 311 patients in pre-TMTC era with treatment results in TMTC era). (DOCX) [file pone.0057719.s003.docx]

**Table S3.** Prognostic factors associated with treatment success for 340 patients (excluding 311 patients in pre-TMTC era with treatment results in TMTC era)

| **Covariate** | **Classification (n)** | **n (%) of treatment success** | **Univariable OR (95% CI)** | **p value** | **Multivariate aOR ^a^ (95% CI)** | **p value** |
| --- | --- | --- | --- | --- | --- | --- |
| Group | TMTC era (290) | 239 (82) | 3.4(1.8-6.4) | <0.001 | 3.8(1.8-7.9) | <0.001 |
|  | Pre-TMTC era (50) | 29 (58) | Reference |  | Reference |  |
| Sex | Male (261) | 204 (78) | 0.8(0.4-1.6) | 0.587 | 1.2(0.6-2.5) | 0.657 |
|  | Female (79) | 64 (81) | Reference |  | Reference |  |
| Aboriginal | Yes (53) | 44 (83) | 1.4(0.6-3.0) | 0.417 | - |  |
|  | No (287) | 224 (78) | Reference |  | - |  |
| BMI | <22 (207) | 155 (75) | Reference |  | Reference |  |
|  | 22~26 (103) | 89 (86) | 2.1(1.1-4.1) | 0.021 | 2.4(1.2-4.9) | 0.019 |
|  | >26 (30) | 24 (80) | 1.3(0.5-3.5) | 0.543 | 1.0(0.3-2.9) | 0.986 |
| Age | <35 (68) | 63 (93) | 7.3(2.7-19.8) | <0.001 | 8.4(2.9-24.7) | <0.001 |
|  | 35~60 (166) | 138 (83) | 2.9(1.6-5.1) | <0.001 | 3.5(1.9-6.6) | <0.001 |
|  | >60 (106) | 67 (63) | Reference |  | Reference |  |
| Alcohol | Yes (56) | 43 (77) | 0.9(0.4-1.7) | 0.683 | - |  |
|  | No (284) | 225 (79) | Reference |  | - |  |
| Diabetics | Yes (106) | 82 (77) | 0.9(0.5-1.5) | 0.656 | - |  |
|  | No (234) | 186 (79) | Reference |  | - |  |
| Hypertension | Yes (61) | 46 (75) | 0.8(0.4-1.5) | 0.472 | - |  |
|  | No (279) | 222 (80) | Reference |  | - |  |
| Hepatitis B | Yes (24) | 20 (83) | 1.4(0.5-4.2) | 0.576 | - |  |
|  | No (316) | 248 (78) | Reference |  | - |  |
| Hepatitis C | Yes (31) | 19 (61) | 0.4(0.2-0.8) | 0.015 | 0.5(0.2-1.2) | 0.102 |
|  | No (309) | 249 (81) | Reference |  | Reference |  |
| Cavitary lesion on CXR | Yes (136) | 104 (76) | 0.8(0.5-1.3) | 0.386 |  |  |
|  | No (204) | 164 (80) | Reference |  |  |  |
| Smear-negative at the time of MDR-TB diagnosis | Negative (135) | 108 (80) | 1.1(0.7-1.9) | 0.667 | - |  |
|  | Positive (205) | 160 (78) | Reference |  | - |  |
| Culture converted before second-line drug | Negative (91) | 76 (84) | 1.5(0.8-2.8) | 0.202 | - |  |
|  | Positive (249) | 192 (77) | Reference |  | - |  |
| Number of first-line drugs to  which isolate is resistant | ≧3 (159) | 115 (72) | 0.5(0.3-0.8) | 0.007 | 0.4(0.2-0.8) | 0.005 |
|  | <3 (181) | 153 (85) | Reference |  | Reference |  |
| ^b^Treatment delay | No (268) | 212 (79) | 1.1(0.6-2.0) | 0.807 | - |  |
|  | Yes (72) | 56 (78) | Reference |  | - |  |
| Patient classification | New (136) | 116 (85) | 3.9(1.2-12.1) | 0.020 | 3.9(1.5-10.4) | 0.007 |
|  | Relapse+ treatment after failure of the first treatment + treatment after default (189) | 143 (76) | 2.1(0.7-6.1) | 0.188 | 2.3(0.9-5.8) | 0.071 |
|  | Treatment after failure of re-treatment (15) | 9 (60) | Reference |  | Reference |  |

^a^Multiple logistic analysis, adjusted with covariates chosen by Akaike information criterion (AIC).

^b^Treatment delay: the lag between sputum collection of MDR-TB and start of second-line drug > 120 days.

Abbreviations: aOR: adjusted odds ratio; BMI: body mass index; CI: confidence interval; CXR: chest radiograph; MDR: multidrug-resistant; OR: odds ratio; TB: tuberculosis; TMTC: Taiwan Multi-drug Resistance Tuberculosis Consortiums.
